# Horner’s Syndrome After Anterior Decompression and Fusion for Cervical Spine Pathologies: Report of Two Cases

**DOI:** 10.7759/cureus.16633

**Published:** 2021-07-26

**Authors:** Tomotaka Umimura, Satoshi Maki, Masao Koda, Takeo Furuya, Seiji Ohtori

**Affiliations:** 1 Orthopedic Surgery, Graduate School of Medicine, Chiba University, Chiba, JPN; 2 Orthopedic Surgery, Faculty of Medicine, University of Tsukuba, Tsukuba, JPN; 3 Orthopedic Surgery, Chiba University Hospital, Chiba, JPN

**Keywords:** horner’s syndrome, anterior cervical discectomy and fusion, ptosis, miosis, iatrogenic complication

## Abstract

Horner’s syndrome is caused by impairment of the sympathetic trunk, resulting in associated ptosis, miosis, and anhidrosis. The cervical sympathetic trunk is sometimes damaged during an anterior approach to the lower cervical spine. We report two cases of Horner’s syndrome after anterior decompression and fusion for lower cervical spine pathologies. Case 1 was in a 58-year-old woman with a herniated C5-6 intervertebral disc presenting myelopathy who underwent anterior cervical discectomy and fusion of C5-6. After the operation, miosis and anhidrosis of the right face occurred, and the symptoms continued for more than 15 years. Case 2 was in a 40-year-old woman whose diagnosis was flexion myelopathy with kyphosis at C5-6 and canal stenosis, so she underwent anterior cervical C5-6 discectomy and fusion of C5-6. Immediately after surgery, ptosis and miosis occurred, which lasted for four months. Horner’s syndrome tends to occur during anterior cervical spine procedures, especially at the lower level, and the syndrome may be transient or irreversible. During an anterior approach to the lower cervical spine, taking care not to damage the sympathetic trunk is important to avoid this complication.

## Introduction

The cervical sympathetic trunk can be damaged during an anterior approach to the lower cervical spine, resulting in Horner’s syndrome with associated ipsilateral ptosis with pupillary constriction, nasal stuffiness, and anhidrosis. It may seem to cause no primary functional impairment, but cosmetic concerns and nasal stuffiness are a discomforting outcome for the patient.

Previous studies have estimated the incidence of Horner’s syndrome to be 0.1%-4.0% after anterior cervical spine surgery [1-4]. Horner’s syndrome as a complication of anterior and anterolateral approaches to the cervical spine may resolve spontaneously [1,5]. However, there are also reports of irreversible symptoms [4].

We present two cases of Horner’s syndrome after anterior cervical spine surgery.

## Case presentation

Case 1

**Figure 1:**
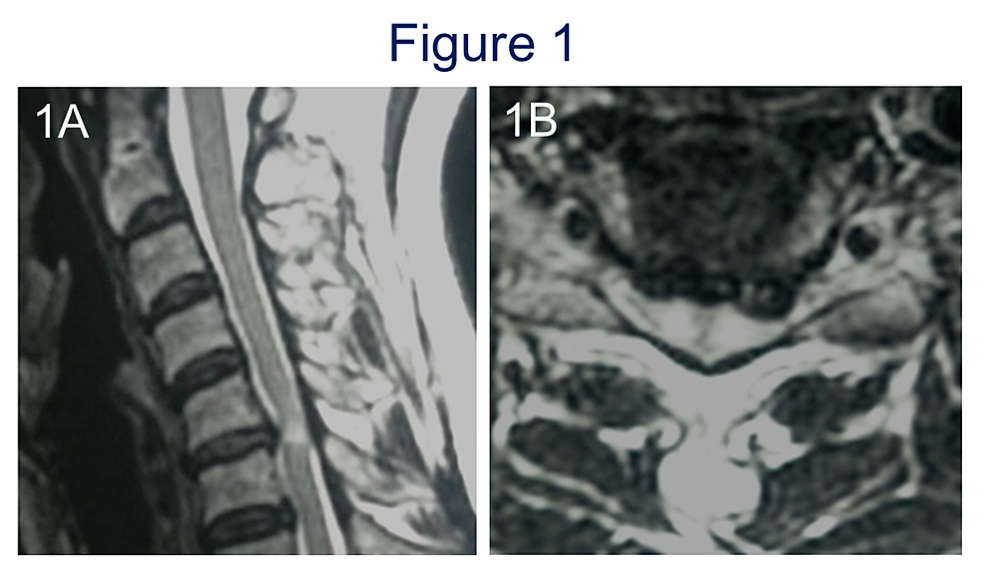
Preoperative T2 MRI sagittal view and axial view at C5-6 of case 1. MRI sagittal view showed canal stenosis at the C5-6 level (A) and axial view revealed a herniated C5-6 intervertebral disc (B).  High signal intensity was seen in the spinal cord at C5-6 disc level.

**Figure 2:**
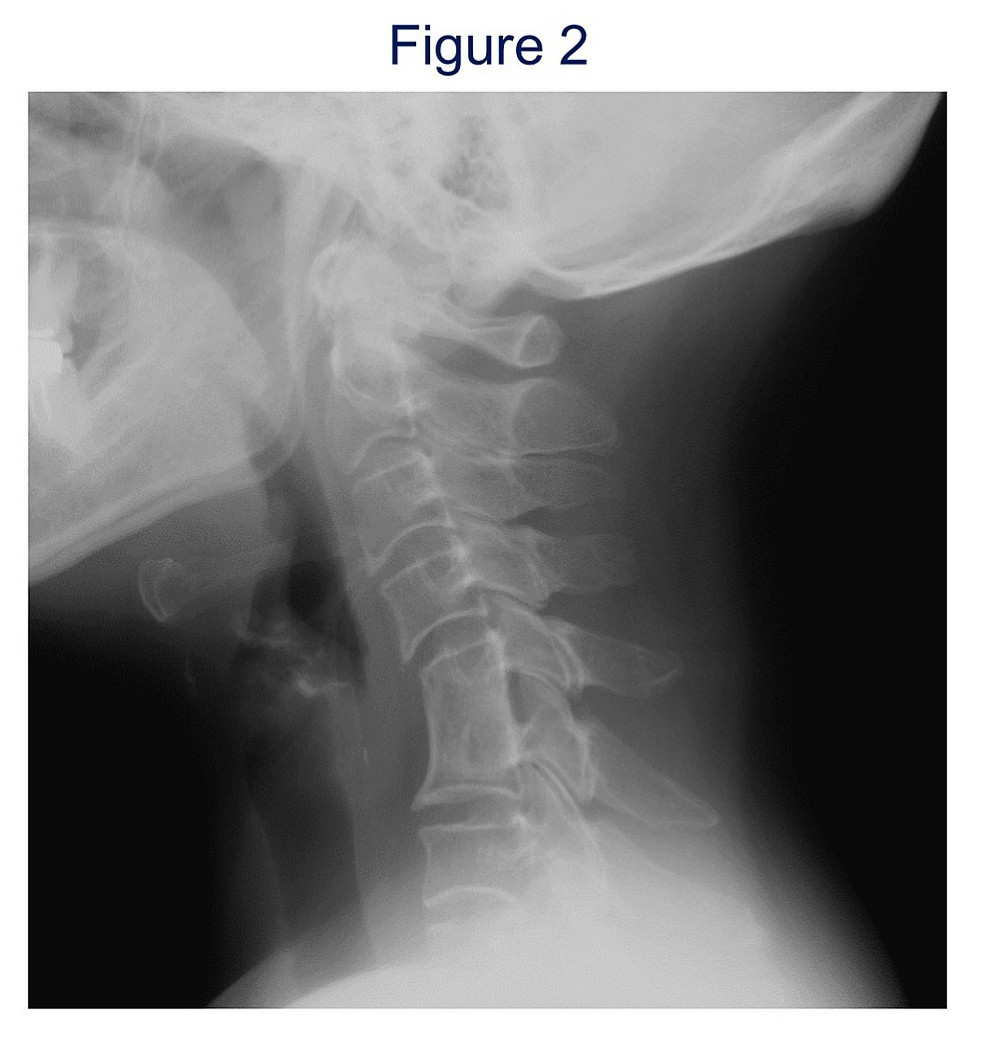
Postoperative lateral radiograph of case 1 (13 years after the surgery). Anterior cervical discectomy and fusion of C5-6 with an autogenous ilium bone graft without instrumentation was performed.

A 58-year-old Japanese woman complained of pain and numbness of both hands with gait disorder. The patient suffered from numbness of her hands and weakness of her right leg for one and a half years. These symptoms were not improved by conservative therapy and became gradually worse, and so she was referred to our hospital. Clinical findings included hyperreflexia, gait disorder, pain, and numbness of both hands. We found a herniated C5-6 intervertebral disc and canal stenosis on MRI (Figure 1), so we performed anterior cervical C5-6 discectomy and fusion of C5-6 (Figure 2). We used the right anterior approach to the vertebral body and harvested autogenous ilium bone for grafting. No complications were observed during the operation. The day after the operation, miosis and anhidrosis of the right face occurred. We made a diagnosis of Horner’s syndrome and treated it conservatively. Preoperative symptoms improved and the patient was discharged six days after surgery, but the symptoms of Horner's syndrome had persisted until the final follow-up for more than 15 years.

Case 2

**Figure 3:**
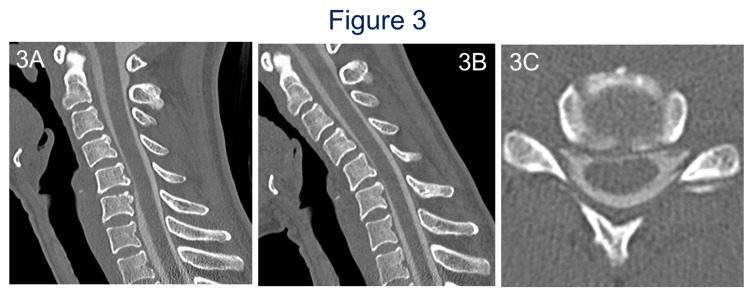
Preoperative CT myelography of case 2. CT myelography showed neutral position sagittal view (A) and anteflexion sagittal view (B), axial view at C5-6 in a flexed position (C) revealing anterior shift of the spinal cord and the spinal cord contacting vertebrae at the C5-6 level with the cervical spine in only flexed position. CT, computed tomography.

**Figure 4:**
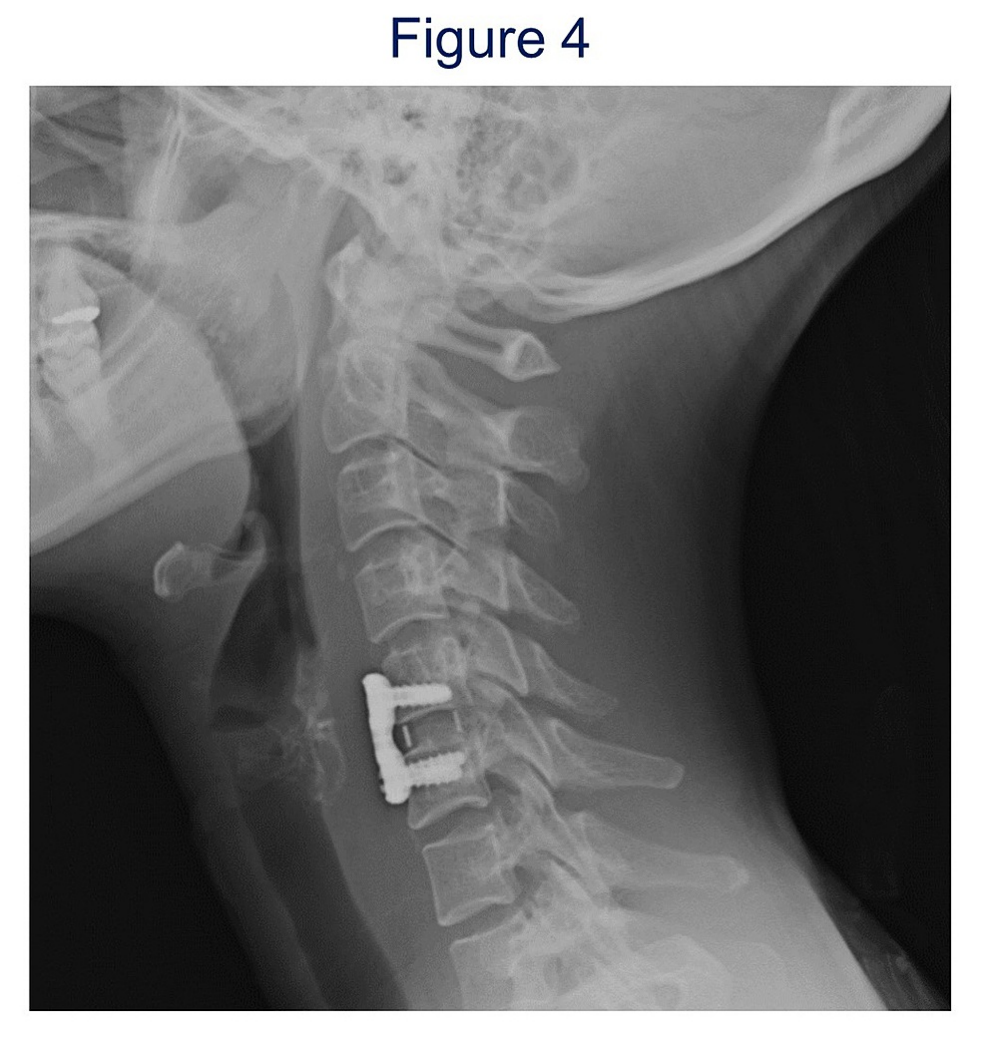
Postoperative lateral radiograph of case 2 (seven years after the surgery). C5-6 anterior fixation with cage and anterior plate was performed.

**Figure 5:**
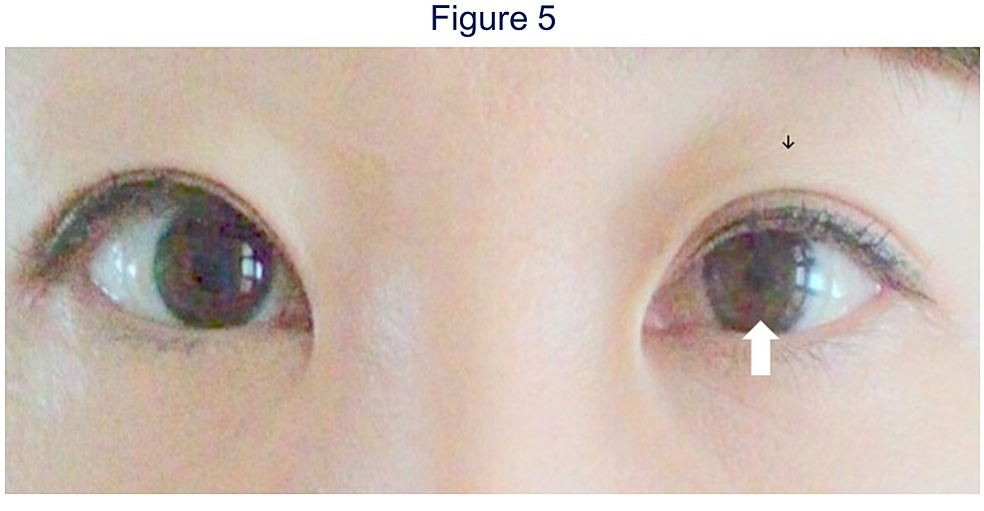
Postoperative picture of case 2. One day after the operation, Horner’s syndrome as left ptosis (black arrow) and miosis (white arrow) appeared.

A 40-year-old Japanese woman complained of muscle weakness of her right hand. She had numbness of her right hand, and motor weakness of extension of her right third and fourth fingers. Manual muscle testing of extension of her right third and fourth fingers were grades 2/5 and 3/5, respectively. She was referred to our hospital and we found kyphotic alignment of her cervical spine. Anterior shifting of the spinal cord and contact with vertebrae at the C5-6 level in a flexed position of the cervical spine were seen on a computed tomography myelogram (Figure 3). We performed anterior cervical C5-6 discectomy and fusion of C5-6. We used a left anterior approach to the vertebral body and fixed it with the polyether ether ketone cage and anterior plate (Figure 4). There was no complication during the operation. One day after the operation, despite the improvement in the weakness of her hand, Horner’s syndrome occurred with associated ptosis, and miosis of her left face (Figure 5). The symptoms in her right fingers improved and the patient was discharged six days after the operation. Four months after surgery the Horner’s syndrome resolved spontaneously.

## Discussion

Horner’s syndrome is a relatively rare complication of anterior surgical approaches to the cervical spine, especially the lower cervical spine. In most cases, it is a temporary deficit and will resolve spontaneously with conservative treatment. However, it could be a permanent complication in some cases. Physicians should be vigilant for this complication during an anterior approach to the lower cervical spine. Horner’s syndrome occurred ipsilateral to the surgical site after anterior C5-6 decompression and fusion in both of our patients. The symptoms were permanent in one patient, while they lasted four months in the second patient.

Horner’s syndrome occurs more frequently after lower anterior cervical spine procedures. Considering the neuroanatomy of the cervical sympathetic trunk, it converges medially descending from upper cervical levels to the lower levels [6]. In a study of 28 adult cadavers (11 male and 17 female), the sympathetic trunk ran diagonally upwards with an average of 10.4 ± 3.8° relative to the midline [7]. Anatomic study demonstrated that the sympathetic trunks were situated 10.6 ± 2.6 mm on average lateral to the medial border of the longus coli muscle. However, they were relatively closer to the medial border of the longus coli muscle at the C6 level than they were at the C3 level. Another study of 30 adult cadavers (23 male and seven female) showed that the sympathetic trunks were situated 11.6 ± 1.6 mm lateral to the medial border of the longus coli muscle, but the distance between the sympathetic trunks was shorter at the lower level than it was at the upper level [8]. The cervical sympathetic trunk can be damaged during an anterior surgical approach to the lower cervical spine or cervical-thoracic junction. In the cases reported here, there were no obvious events that damaged the sympathetic trunk during surgery, but it was possible that the sympathetic trunk was damaged during retraction. Both patients underwent anterior decompression and fusion of C5-6, causing Horner's syndrome. These cases are compatible with previous reports.

The time it takes for resolution of Horner’s syndrome after anterior and anterolateral approaches to the cervical spine is controversial. Although in some reports the symptoms spontaneously resolved within several months [1,5], others report that the symptoms were irreversible [4]. Considering our case 1, the patient has suffered from miosis and anhidrosis for more than 15 years. In case 2, the patient’s symptoms disappeared spontaneously four months after the operation.

Much care should be taken not to cause Horner’s syndrome while approaching the anterolateral part of the cervical vertebrae, especially at the lower cervical levels. Retracting the longus coli muscle and soft tissues laterally, and extending dissection to the longus coli muscle or stripping this muscle, may cause sympathetic damage producing Horner’s syndrome. To avoid injury of the cervical sympathetic trunks, we suggest placing the blunt tip of the retractor securely beneath, rather than on, the surface of the longus coli muscle during the approach to the lower cervical spine [7]. Attention should also be paid when extensively dissecting over or below the longus coli muscle.

## Conclusions

We experienced two cases with Horner’s syndrome after anterior decompression and fusion at C5-6 and were treated conservatively. Symptoms persisted permanently in the first case and resolved spontaneously within four months in the second case. Horner’s syndrome is a relatively rare complication of anterior cervical spine surgery, but may occur especially in the lower cervical spine. The symptoms are transient in the majority of cases, but may sometimes be an irreversible complication. Surgeons should take care to remain in the midline during an anterior approach to the lower cervical spine to avoid this complication.
